# Cross-cultural adaptation of the delphi definitions of low back pain prevalence (German DOLBaPP)

**DOI:** 10.1186/1471-2474-15-397

**Published:** 2014-11-25

**Authors:** Marja Leonhardt, Falk Liebers, Clermont E Dionne, Ute Latza

**Affiliations:** Berlin School of Public Health at the Charité, Seestr. 73, D-13347 Berlin, Germany; Federal Institute for Occupational Safety and Health (BAuA), Noeldnerstr. 40/42, D-10317 Berlin, Germany; Axe Santé des populations et pratiques optimales en santé du Centre de recherche FRQ-S du CHU de Québec, 1050, chemin Ste-Foy, Québec City, QC G1S 4L8 Canada

**Keywords:** Questionnaire, Validation, Low back pain, Pre-test, Cross-cultural adaptation, Prevalence, Site, Symptoms, Duration, Functional limitation

## Abstract

**Background:**

Assessed dimensions of low back pain (LBP) vary in prevalence studies. This may explain the heterogeneity in frequency estimates. To standardize definitions of LBP, an English consensus with 28 experts from 12 countries developed the “Delphi Definitions of Low Back Pain Prevalence” (DOLBaPP). The optimal definition and the shorter minimal definition with the related questionnaires for online, paper, and face-to-face use and telephone surveys are suitable for population-based studies. The definitions have to be adapted to different languages and cultures to provide comparable frequency estimates. The objective was to culturally adapt and pre-test the English definitions and corresponding Delphi DOLBaPP questionnaire forms into German.

**Methods:**

The German DOLBaPP adaptation was conducted using the systematic approach suggested by Beaton et al. A pre-test of the Delphi DOLBaPP optimal paper questionnaire including an additional evaluation form was conducted in a sample of 121 employees (mainly office workers). In order to evaluate the comprehensibility, usability, applicability, and completeness of the adapted questionnaire, response to the questionnaire and 6 closed evaluation questions were analyzed descriptively. Qualitative methods were used for the 3 open questions of the evaluation form.

**Results:**

The cultural adaptation of the DOLBaPP for a German-speaking audience required little linguistic adaptation. Conceptual equivalence was difficult for the expression “low back pain”. The expert committee considered the face validity of the pre-final version of the related Delphi DOLBaPP questionnaires as good. In the pre-test, most participants (95%) needed less than 5 minutes to fill in the optimal Delphi DOLBaPP questionnaire. They were generally positive regarding length, wording, diagram, and composition. All subjects with LBP (n = 61 out of 121 – 50.4%) answered the questions on functional limitation, sciatic pain, frequency and duration of symptoms as well as pain severity.

**Conclusion:**

The results indicate that the cross-cultural German adaptation of the DOLBaPP Definitions and the corresponding questionnaires was successful. The definitions can be used in epidemiological studies to measure the prevalence of LBP. Some critical issues were raised regarding the general features of the Delphi DOLBaPP questionnaires. Future research is needed to evaluate these instruments.

**Electronic supplementary material:**

The online version of this article (doi:10.1186/1471-2474-15-397) contains supplementary material, which is available to authorized users.

## Background

Among the assessment instruments available for low back pain (LBP) as outcome in epidemiological studies, there is large heterogeneity both internationally and nationally [1–3]. In a systematic review of 165 studies from 54 countries, the mean one-month prevalence of LBP was 30.8% with a standard deviation of 12.5% [3]. The standard deviation for the one-year prevalence was even larger [3].

Within a modified Delphi method, 28 experts from 12 countries agreed on standardized items – the “Delphi Definitions of Low Back Pain Prevalence” (DOLBaPP). The standardized definitions correspond to questionnaires that can be used in prevalence studies [4]. The minimal definition has one question on pain characteristics (site, symptoms, and time frame), and a second question on functional limitation due to LBP. The optimal definition has five more questions covering frequency and duration of symptoms, pain intensity, sciatica, and exclusions that can be adapted to different needs. The minimal definition is proposed for use in studies with time or space constraints [5]. Both definitions were developed in English and can be openly accessed [6]. In German speaking populations, different instruments are used to measure the prevalence of LBP [7–21]. This heterogeneity makes it difficult to compare or summarize results from different studies. The study objective was to perform a cross-cultural adaption of the definitions and the related Delphi DOLBaPP questionnaires for German-speaking adults. A specific feature of the German language is the lack of an equivalent translation for LBP.

## Methods

**Figure 1:**
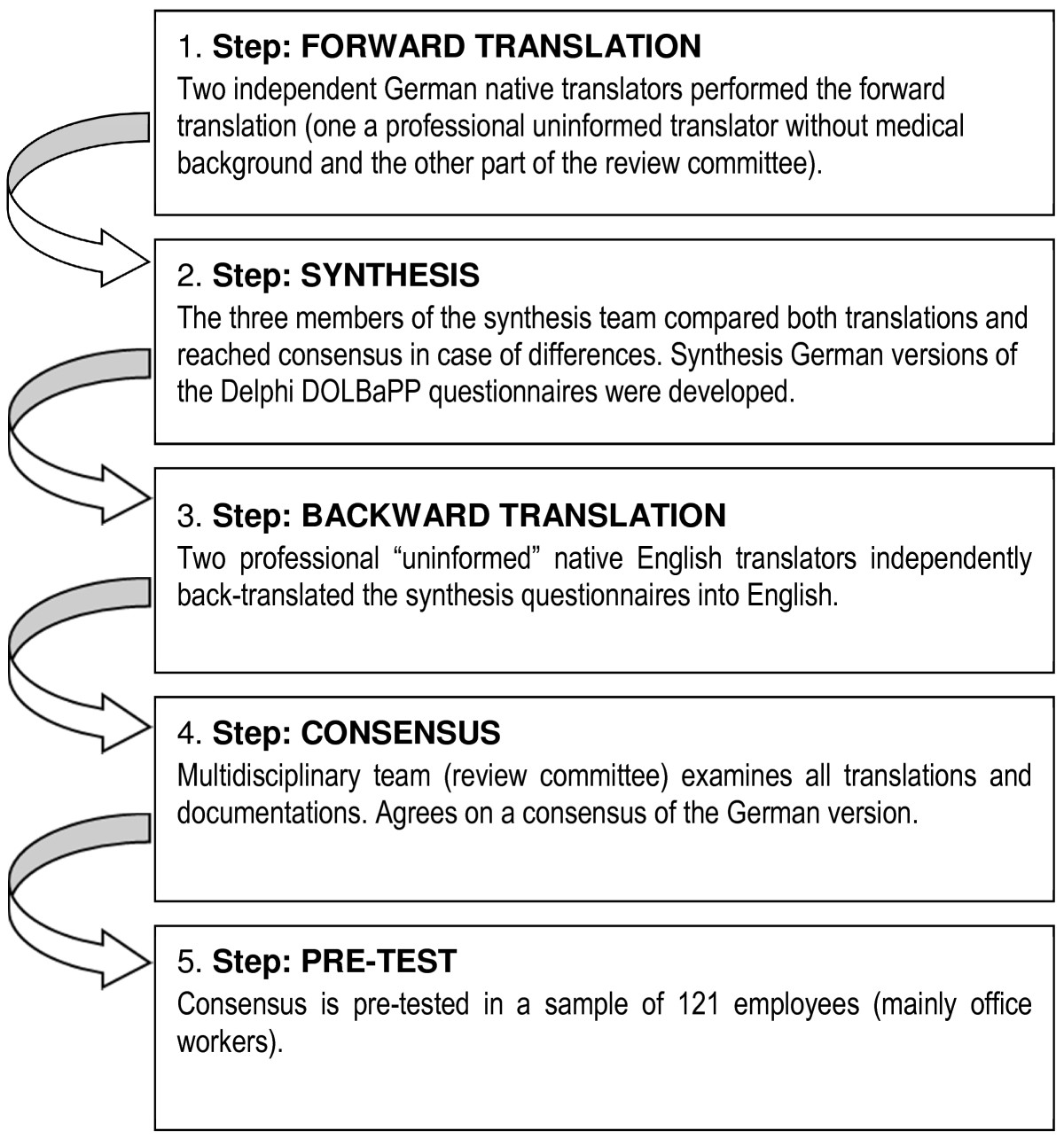
**Forward-Backward-Translation method (adapted from Beaton et al. [**[23]**]).**

The Delphi DOLBaPP Definitions and questionnaires were translated to German following internationally recommended methodology (see Figure 1) [5, 22, 23]. As the minimal two-item definition is included in the optimal seven-item definition, there was no separate adaptation for the minimal version.

Consolidation of all translated versions into a pre-final optimal questionnaire, for pre-test, was carried out with a multidisciplinary review committee (ML, FL, MT, and UL). Moreover, a bilingual English native speaker without linguistic or medical background was consulted, to check the suitability for daily use of the translations. Further, an additional opinion of a German native speaker living in the United States was requested.

The review committee decided that in case of an agreement of the diction in both backward translations with the English original, the equivalence in German would be quoted as a consensus. Furthermore they collected cases of doubts, which were addressed by the English translators. The refreshed input of the translators generated the consensus of the German version of the Delphi DOLBaPP optimal questionnaires. It was necessary to adapt some wordings for German speaking countries, e.g. there are only medical terms, but no equivalent expression for daily use of “low back pain” in German.

### Pre-test

To evaluate the equivalence and comprehensibility of the translated version of the optimal questionnaire (form O3), a pre-test was conducted [21, 24, 25] among all employees of a federal employer (172 employees aged 18 to 65 years in a large German city). The objective was to check the suitability of the adapted DOLBaPP German version of the optimal questionnaire within German speaking countries [26].

The pre-test was planned and conducted in accordance with the Helsinki Declaration of 1996, and the German Federal Data Protection Act. The data protection commissions of the Charité (Behördliche Datenschutzbeauftragte, Charité - Universitätsmedizin Berlin) and of the participating federal employer (Behördlicher Datenschutzbeauftragter) approved the procedures. Written informed consent for participation was obtained before the survey. Employees received written information via electronic mail about main features of the study, their contribution to the prevention of work-related LBP, and the data protection procedures. Employees participated voluntarily. They were informed that they accepted the conditions of the study and their participation by anonymously returning the questionnaire in a closed envelop via internal mail. The questionnaire and evaluation form with information sheet were distributed via internal mail to the employees. In order to increase response, information on age and gender that might hinder confidentiality protection was not requested.

A total of 121 employees participated (70.3% of those approached) and filled in the DOLBaPP German version of the questionnaire and an evaluation form.

### Evaluation of the pre-test

Quantitative data were described using PASW Version 18.0.0 for Mac (SPSS) (distribution, mean, congruence and missing values). Chronic LBP was defined as pain that lasted for three months or more [27]. For qualitative analysis of the three open questions of the evaluation form, the answers were tagged and categories were built [28]. The results of the Delphi DOLBaPP optimal questionnaire were linked with the results of the evaluation form. The comments from the synthesis and the review committee were compared with the comments of the participants.

## Results

### The cross-cultural adaptation process

After the structured forward backward translation process, the review committee agreed on consensus German definitions of the DOLBaPP and the related questionnaire forms for paper, online or face-to-face use (optimal definition see Table 1) and telephone surveys. The synthesis team and the review committee recorded similar challenging idiomatic issues in the translation: in general the German language has only an expression for “pain in the lower back” and no generally accepted expression for “low back pain”. Thus, conceptual discrimination of the two expressions is not possible. As a decision, “pain in the lower back” was used throughout the questionnaire. For item 1, the personal pronoun “your” in the expression “your lower back” is rather unusual in the German language in this context. For idiomatic equivalence it was changed to “the lower back”. The expression “diagram” proved to be difficult to translate, and was translated from “*Bild*” to “*Abbildung*” in the German version referring to the schematic presentation in the questionnaire. For item 2, the original expression “bad” was changed in the backward translation process to “severe” because the German synonym for bad pain is too colloquial in the German language. The backward translators suggested that “bad pain” and “severe pain” had the same meaning. The expression “go” in items 3 and 4 was changed to “radiate” because “radiate” better expresses the idea in German. Furthermore in colloquial German there is no differentiation between the tenses “has this pain gone” and “did this pain go”. In the answer options of item 5 (pain frequency) the team could not detect whether “on most days “meant pain e.g. in 60 percent (“*an den meisten Tagen*”) or 90 percent of the days (“*an fast allen Tagen*”). The expression “how long was it since you had” in item 6 translates into a very complicated German sentence. Thus a German version similar to “when was the last time that you had” was chosen. The distinction between “last 4 weeks” and “past 4 weeks” was considered irrelevant. Comparisons with other German questionnaires (“last 3 months”, [17] “in the last week”, [19] “last 7 days”, [18] “last week” [16]) favor the chosen translation. For item 7, the teams suggested to consider the expression “…a scale from 0 to 10” instead of “…a scale of 0 to 10” in the English version. The distinction between “indicate … on a scale …” and “give … on a scale …” was also considered irrelevant.

**Table 1:** **Response in the pre-test of the optimal Delphi Definitions of Low Back Pain Prevalence (DOLBaPP) German language version (n = 121)**

Item original English (German adaption)	Number of respondents			
	**Yes** (Ja)	**No** (Nein)		
**Q1- In the last 4 weeks, have you had pain in your lower back? Please ignore pain caused by menstruation or by an illness accompanied by fever.** (Hatten Sie in den *letzten 4 Wochen* Schmerzen im unteren Rücken (im Bereich, der in der Abbildung markiert ist)? Bitte ignorieren Sie Schmerzen, wenn sie im Zusammenhang mit Fieber oder der Menstruation aufgetreten sind.)	61	60		
**Q2- If yes, was this pain bad enough to limit your usual activities or change your daily routine for more than one day?** (Wenn ja, waren diese Schmerzen so stark, dass Sie länger als einen Tag Ihre üblichen Tätigkeiten eingeschränkt oder Ihre alltäglichen Aktivitäten verändert haben?)	15	66		
**Q3- In the last 4 weeks, have you had pain that goes down the leg?** (Hatten Sie in den *letzten 4 Wochen* Schmerzen, die bis ins Bein ausstrahlten?)	17	102		
**Q4- If yes, has this pain gone below the knee?** (Wenn ja, strahlten diese Schmerzen bis unterhalb des Knies aus?)	10	53		
	**On some days** (An einigen Tagen)	**On most days** (An den meisten Tagen)	**Every day** (Jeden Tag)	
**Q5- If you had pain in your lower back in the last 4 weeks, how often did you have the pain?** (Wenn Sie in den *letzten 4 Wochen* Schmerzen im unteren Rücken hatten, wie oft hatten Sie diese Schmerzen?)	50	9	3	
	**Less than 3 months** (Weniger als 3 Monate)	**3 months or more, but less than 7 months** (3 Monate oder mehr, aber weniger als 7 Monate)	**7 months or more, but less than 3 years** (7 Monate oder mehr, aber weniger als 3 Jahre)	**3 years or more** (3 Jahre oder mehr)
**Q6- If you had low back pain in the last 4 weeks, how long was it since you had a whole month without any low back pain? (Please tick only one box).** (Wenn Sie in den *letzten 4 Wochen* Schmerzen im unteren Rücken hatten, wie lange ist es her, dass Sie einen ganzen Monat lang gar keine derartigen Schmerzen hatten? (Bitte kreuzen Sie nur ein Kästchen an)).	23	16	9	13
	**Mean (scale 0-10) and Standard deviation**	**Minimum, Maximum and Median**		
**Q7- If you had low back pain in the last 4 weeks, please indicate what was the usual intensity of your pain on a scale of 0 to 10, where 0 means “no pain” and 10 means “the worst pain imaginable”. (Please circle your answer).** (Wenn Sie in den *letzten 4 Wochen* Schmerzen im unteren Rücken hatten, geben Sie bitte auf einer Skala von 0 bis 10 die übliche Intensität der Schmerzen an. Dabei bedeutet 0 „keine Schmerzen” und 10 „stärkster vorstellbarer Schmerz”. (Bitte kreisen Sie die Antwort ein).	Mean = 3,86	Min = 1		
	SD = 1,66	Max = 10		
		Median = 4		
	(n = 59)	(n = 59)		

### Pre-test of the Delphi DOLBaPP optimal questionnaire

About half of the participants who filled in the pre-final Delphi DOLBaPP optimal questionnaire (n = 61 out of 121 – 50.4%) had had pain in the lower back in the last four weeks (see Table 1). All employees who suffered from LBP also answered the question, if the pain was bad enough to limit their usual activities or change their daily routine. About 24.6% (n = 15) of those with LBP answered positively, corresponding to 12.4% with LBP in the last four weeks combined with functional limitation. Of the participants who had had LBP in the last four weeks, 62.3% suffered from chronic LBP. If only the participants with LBP were included (n = 61), the results of the question about pain intensity were nearly normally distributed. Only 2 participants with LBP did not answer this question.

Item 2 – the question about limitation due to LBP – was answered by 20 participants with “no”, although they had not reported LBP. In item 3 (determination of sciatica), 102 answers with “no” were registered, even though this item is only supposed to be answered by participants who had had LBP in the last four weeks. Furthermore 17 participants stated that they were suffering from pain that goes down the leg; thus 17 participants should have answered the following question, whether the pain goes below the knee. Nevertheless the pre-test did not show 105 missing entries, as expected, but only two.

The full scale of the answer categories in item 5, 6 and 7 was utilized. All 61 employees with LBP answered item 5 (pain frequency) and item 6 (duration of LBP). Six participants who stated that they did not have LBP indicated in item 7 (pain intensity) “0 = no pain”. However, only participants with LBP were supposed to answer this item. This is implicated by the formulation “if yes …” of the repetition “If you had pain in your lower back in the last 4 weeks …” Altogether there were no implausible answers, apart of one exception: One person stated in item 1 that he/she had no LBP, but answered in item 7 that the pain was very severe (9/10).

### Results of the evaluation form

Every study participant completed the evaluation form. The majority answered the open questions. Most of the study participants (95.0%) needed less than 5 minutes to fill in the Delphi DOLBaPP optimal questionnaire. The majority (83.5%) considered the length of the Delphi DOLBaPP optimal questionnaire as appropriate; the questionnaire was too short and not detailed enough for 16 participants. They suggested adding more items concerning other health aspects. The majority (77.7%) considered the wording of the questions as understandable. The diagram in the first item of the questionnaire was clear for most of the participants. About 26% of the participants (n = 31) were not satisfied with the composition of the questionnaire. In the evaluation form, 41 respondents noted that the guidance in the DOLBaPP optimal questionnaire was not optimal. They commented that further advice for a person who indicated in item 1 that he or she did not suffer from LBP, was not to fill in the remaining questionnaire was missing. Seven respondents commented that it was not clear which sort of pain was meant in item 3. Other respondents noted that it was difficult to discriminate whether the pain went down the leg or not. Some respondents were not satisfied with the answer options in item 5. They missed an answer option for somebody who had LBP only on one day. About eight respondents thought that the wording of item 6 was ambiguous. They suggested to remove the negation in the question and to shorten the sentence. Also the answer options in the question were implausible for some of the respondents. Four participants noted that in item 7 the zero in the scale (which means no pain) did not make sense, as it was assumed that the interviewee had LBP. Other four respondents noted that the diagram in item 1 was not appropriate as it was not obvious whether the manikin is displayed from the back or the front side.

Altogether, 35 suggestions were made for supplementary aspects in the questionnaire, most respondents gave various suggestions. For nine respondents, it would be important to ask for the reason of LBP, specifically in which (working) situation they occurred. Seven respondents would ask additionally for neck or shoulder pain prevalence. Whether medicine was taken or a doctor was seen for LBP were more suggestions for additional questions the respondents made. Other suggestions were: whether housework, leisure time or the job is limited by the LBP; physical activity, first date of LBP; time of occurrence of LBP; pre-existing illness; secondary consequences of LBP (e.g. sleep deficit, sick-leave etc.).

## Discussion

The objective of the study was to conduct a cross-cultural adaptation of the definitions and related Delphi DOLBaPP questionnaires for German-speaking countries using a systematic multi-step approach [23]. This resulted in German versions of the Delphi DOLBaPP Definitions that are considered equivalent to the original English versions.

The culturally adapted Delphi DOLBaPP optimal questionnaire for face-to face interviews and paper or online administration was pretested. The performed pre-test gave no evidence for a linguistic inconsistency, but some hints for a linguistic optimization of the translated German version of the DOLBaPP optimal questionnaire: Regarding the answer options in item 5 on pain duration, an additional answer option e.g. “on one day” could be considered. Furthermore the wording of the question in item 6 on duration and recurrence seemed complicated for some participants, as it is a multi-clause sentence. One reason might be that the German translation of the expression “low back pain” is quite long. In fact, the German translation of this expression is a general problem. However, the expression “low back pain” is generally not used consistently in every single item of the questionnaire: item 3 asks only if one had “pain”. For the participants of the pre-test, it was not clear whether the question referred to LBP or pain in general. This wording might bias the results as participants who answered this item with “yes” might suffer from pain which had no origin in the back. In addition, the expert committee felt that two concepts were covered by item 6: chronicity and new episode. Participants of the pre-test did not comment this exactly but criticized the answer options in which not every participant found himself or herself. It might be considered whether it is sensible to use this item to determine chronicity, as people with chronic LBP could also be pain-free for a longer period (which might be longer than 4 weeks).

No representative sample was chosen for the pre-test. However, the prevalence of LBP in the last four weeks (50.4%) in the pre-test was within the range of estimates for other office workers e.g. [29]. In particular, the prevalence of LBP with functional limitation according to the minimal definition of the DOLBaPP (12.4%) was comparable to the one-month prevalence of disabling LBP among an English speaking population (office workers in the United Kingdom [30]). Further validation of the instruments within larger population-based studies or within blue-collar workers is warranted.

The pre-test gives interesting evidence for the general design of the Delphi DOLBaPP questionnaires, irrespective of language: The structure of the optimal questionnaire is not ideal as people with no LBP have to read every single question of the assessment instrument although they could stop after the first question. A simple instruction explaining that participants who answer ‘no’ to question 1 can ignore the following questions was regarded more user-friendly. After consideration of further external expertise, a modification of the optimal questionnaire is suggested (see Additional file 1). This instruction also reduces the construction of the following items. In particular, the first half sentence of the question in item 6, can be dropped.

Further general aspects of the Delphi DOLBaPP questionnaires relate to item 7, and the diagram. The scale in item 7 was used by nearly all pre-test participants, although the zero on the scale (= no pain) does not make sense considering the question asks for the intensity of the LBP in the last 4 weeks. It might be considered to change the label of the endpoint zero in the scale into “very little pain”. Another consideration is to start the questionnaire with this item. In this case, a little modification (e.g. omit “if you had pain in the last 4 weeks”) of the question would be necessary. Furthermore people with no LBP in the last 4 weeks could tick “zero” on the scale. The diagram irritated some pre-test participants. A modern and larger diagram of a unisex manikin in which the lower back is highlighted might make it clearer which area of the body the questionnaire is about. Further evaluations of the questionnaires for telephone surveys may be warranted as well as adaption of the questionnaires for online surveys.

The use of the Delphi DOLBaPP Definitions and related questionnaires will allow for further investigation of the test-retest-reliability (reproducibility) and the validity, particularly regarding different study populations. In order to better compare the results from different studies, the influence of the mode of administration on the frequency of pain also needs to be described (e.g. within an epidemiological study on work-related musculoskeletal complaints or within a general health examination) [31]. Moreover, further development of population-based questionnaires on LBP prevalence might integrate related aspects such as the definition of the recurrence of an episode of LBP. With a similar Delphi approach, Stanton et al. gained an English consensus on LBP recurrence in order to enable comparison between trials evaluating treatment of LBP [32]. Thus, the standardization of LBP prevalence in epidemiological studies should be considered a process. Generally, the experience with the standardization process regarding LBP may also stimulate standardized outcome definitions of pain in other regions of the musculoskeletal system for use in epidemiological studies.

## Conclusions

The Delphi DOLBaPP optimal questionnaires are based on an internationally consented short and basic definition that gives evidence of appearance, duration, frequency and intensity of LBP as well as restrictions due to LBP without building scores. The questionnaires and the shorter minimal definitions can be used in population-based studies. The definitions were successfully adapted to German adults following an internationally recommended methodology. Pre-testing of the Delphi DOLBaPP optimal questionnaire for paper use was performed with 121 mainly white collar employees aged 18 to 65 years. The German versions of the Delphi DOLBaPP questionnaires required only little linguistic adaptation. Critical issues arose not due to the cross-cultural adaptation but to some aspects of the optimal instrument in general (construction and selected items). The authors assume that this does not only apply to the German version of the Delphi DOLBaPP optimal questionnaires, but also to the English original version and other cultural adaptations. Further investigation on the reliability and validity of the instrument is warranted.

## Electronic supplementary material

Additional file 1:Delphi Definitions of Low Back Pain Prevalence (DOLBaPP). Suggested modification for paper questionnaires of the optimal questionnaire (Form O3) German Language Version. (PDF 20 KB)

Below are the links to the authors’ original submitted files for images.

Authors’ original file for figure 1

## Abbreviations

BAuA: Bundesanstalt für Arbeitsschutz und Arbeitsmedizin [Federal Institute for Occupational Safety and Health]
DOLBaPP: Delphi definitions of low back pain prevalence
LBP: Low back pain.
