# Supplementary material for: Cross-cultural adaptation of the delphi definitions of low back pain prevalence (German DOLBaPP)
Source: BMC Musculoskelet Disord. 2014 Nov 25;15:397. doi: 10.1186/1471-2474-15-397 (PMC4258005; doi:10.1186/1471-2474-15-397)
Supplement: Supplementary file 1 — Additional file 1:Delphi Definitions of Low Back Pain Prevalence (DOLBaPP). Suggested modification for paper questionnaires of the optimal questionnaire (Form O3) German Language Version. (PDF 20 KB) [file 12891_2014_2340_MOESM1_ESM.pdf]

Additional file 1

Delphi Definitions of Low Back Pain Prevalence (DOLBaPP). Suggested modification of the optimal questionnaire (Form O3) German Language Version

The example for paper questionnaires is built from the items presented in Table 1. The optimal definition is made up from the minimal definition of low back pain with one question on site, symptoms, and time frame (item 1), and a second question on severity (item 2). For the optimal definition, further questions covering frequency (item 5), duration (item 6), severity (item 7), sciatica (items 3, and 4), and exclusions (see Table 1) are added that can be adapted to different needs as described by Dionne et al. (2008).

Additional file 1

Delphi Definitions of Low Back Pain Prevalence (DOLBaPP). Suggested modification for paper questionnaires of the optimal questionnaire (Form O3) German Language Version

Delphi Fragebogen DOLBaPP

F 1 - Hatten Sie in den <u>letzten 4 Wochen</u> Schmerzen im unteren Rücken (im Bereich, der in der Abbildung markiert ist)?

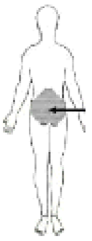

UNTERER RÜCKEN

Ja □ Nein □ ***Wenn nein, bitte weiter mit Frage XY/ gehen Sie bitte zum Ende der Befragung***

F 2 - Wenn ja, waren diese Schmerzen so stark, dass Sie länger als einen Tag Ihre üblichen Tätigkeiten eingeschränkt oder Ihre alltäglichen Aktivitäten verändert haben?

Ja □ Nein □

F 3 - Hatten Sie in den <u>letzten 4 Wochen</u> Schmerzen, die bis ins Bein ausstrahlten?

Ja □ Nein □

F 4 - Wenn ja, strahlten diese Schmerzen bis unterhalb des Knies aus?

Ja □ Nein □

F 5 - Wie oft hatten Sie in den <u>letzten 4 Wochen</u> Schmerzen im unteren Rücken?

□ An einigen Tagen □ An den meisten Tagen □ Jeden Tag

F 6 - Wie lange ist es her, dass Sie einen ganzen Monat lang gar keine Schmerzen im unteren Rücken hatten? (Bitte kreuzen Sie nur ein Kästchen an).

**Weniger als 3 Monate**	□
**3 Monate oder mehr, aber weniger als 7 Monate**	□
**7 Monate oder mehr, aber weniger als 3 Jahre**	□
**3 Jahre oder mehr**	□

F 7 - Geben Sie bitte auf einer Skala von 0 bis 10 die übliche Intensität der Schmerzen im unteren Rücken in den <u>letzten 4 Wochen</u> an. Dabei bedeutet 0 „keine Schmerzen“ und 10 „stärkster vorstellbarer Schmerz“. (Bitte kreisen Sie die Antwort ein).

0	1	2	3	4	5	6	7	8	9	10
**Keine Schmerzen**										**Stärkste vorstellbare Schmerzen**

© Université Laval, 2010, Clermont Dionne, URESP

© Bundesanstalt für Arbeitsschutz und Arbeitsmedizin, Ute Latza 2014 for the modification of the German adaptation, 2013

Die Abbildung wurde zuerst in: Kuorinka I, Jonsson B Kilbom A et al. Standardised Nordic questionnaires for the analysis of musculoskeletal symptoms. *Applied Ergonomics* 1987, 18(3):233-7 veröffentlicht und wird mit Genehmigung des Herausgebers verwendet.
