# Supplementary material for: Cross-cultural adaptation of the delphi definitions of low back pain prevalence (German DOLBaPP)
Source: BMC Musculoskelet Disord. 2014 Nov 25;15:397. doi: 10.1186/1471-2474-15-397 (PMC4258005; doi:10.1186/1471-2474-15-397)
Supplement: Supplementary file 2 — Authors’ original file for figure 1 [file 12891_2014_2340_MOESM2_ESM.pdf]

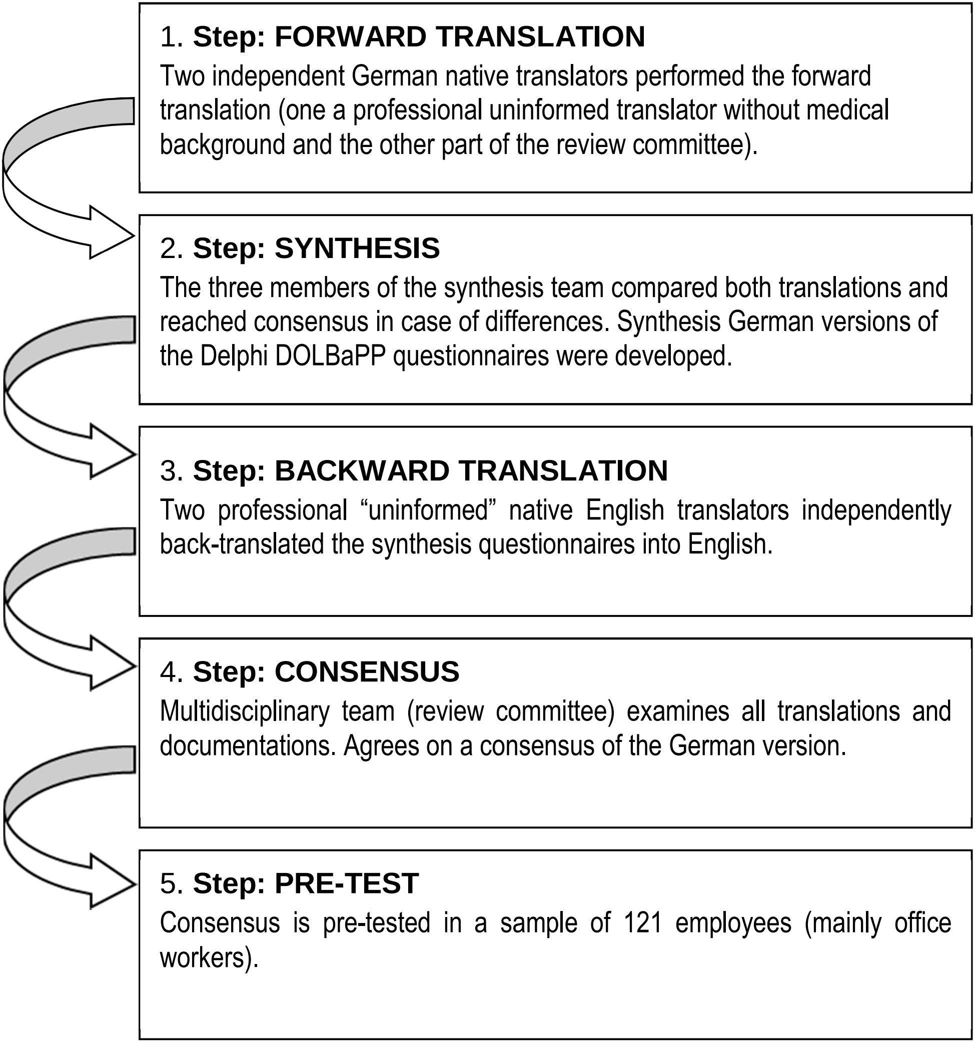
1. Step: FORWARD TRANSLATION
Two independent German native translators performed the forward translation (one a professional uninformed translator without medical background and the other part of the review committee).
2. Step: SYNTHESIS
The three members of the synthesis team compared both translations and reached consensus in case of differences. Synthesis German versions of the Delphi DOLBaPP questionnaires were developed.
3. Step: BACKWARD TRANSLATION
Two professional “uninformed” native English translators independently back-translated the synthesis questionnaires into English.
4. Step: CONSENSUS
Multidisciplinary team (review committee) examines all translations and documentations. Agrees on a consensus of the German version.
5. Step: PRE-TEST
Consensus is pre-tested in a sample of 121 employees (mainly office workers).
